# Hospital Surgical Volume, Utilization, Costs and Outcomes of Retroperitoneal Lymph Node Dissection for Testis Cancer

**DOI:** 10.1155/2012/189823

**Published:** 2012-04-09

**Authors:** Hua-yin Yu, Nathanael D. Hevelone, Sunil Patel, Stuart R. Lipsitz, Jim C. Hu

**Affiliations:** ^1^Division of Urology, Brigham and Women's Hospital, Harvard Medical School, 1153 Centre Street, Suite 4420, Boston, MA 02130, USA; ^2^Center for Surgery and Public Health, Brigham and Women's Hospital, Harvard Medical School, 1153 Centre Street, Suite 4420, Boston, MA 02130, USA; ^3^Department of Urology, David Geffen School of Medicine at UCLA, Los Angeles, CA 90095, USA

## Abstract

*Objectives*. Retroperitoneal lymph node dissection (RPLND) outcomes for testis cancer originate mostly from single-center series. We characterized population-based utilization, costs, and outcomes and assessed whether higher volume affects outcomes. *Methods and Materials*. Using the US Nationwide Inpatient Sample from 2001–2008, we identified 993 RPLND and used propensity score methods to assess utilization, costs, and inpatient outcomes based on hospital surgical volume. *Results*. 51.6% of RPLND were performed at hospitals where there were two or fewer cases per year. RPLND was more commonly performed at large urban teaching hospitals, where men were younger, more likely to be white and earning incomes exceeding the 50th percentile (all *P* ≤ .05). Higher hospital volumes were associated with fewer complications and more routine home discharges (all *P* ≤ .047). However, higher volume hospitals had more transfusions (*P* = .004) and incurred $1,435 more in median costs (*P* < .001). Limitations include inability to adjust for tumor characteristics and absence of outpatient outcomes. *Conclusions*. Sociodemographic differences exist between high versus low volume RPLND hospitals. Although higher volume hospitals had more transfusions and higher costs, perhaps due to more complex cases, they experienced fewer complications. However, most RPLND are performed at hospitals where there were two or fewer cases per year.

## 1. Introduction

Testis cancer is the most common malignancy among US men aged 18–34 years. The overall incidence of germ cell tumors has risen since 1973, and the annual increase in the incidence of nonseminomatous germ cell tumors (NSGCT-) has been 2% per year [1, 2]. Retroperitoneal lymph node dissection (RPLND) is used for the treatment of men with stage I NSGCT, low volume stage II disease, and in the postchemotherapy setting to eradicate residual mass. However, RPLND utilization has decreased since 1988 due to the increased use of surveillance and primary chemotherapy [3].

Due to the relative low incidence of testis cancer compared with other genitourinary malignancies, most reports of RPLND use and outcomes originate from high volume tertiary referral centers, and there is an absence of population-based RPLND outcomes data. While testis cancer mortality and RPLND use rates have been reported from the U.S. Surveillance Epidemiology and End Results (SEER) cancer registry [3], there is currently no way to link pathologic data to nationally representative administrative datasets such as Medicare, due to the age limitation of this patient population. Moreover, while volume outcomes effects have been characterized for oncologic procedures, leading to advocacy for selective referral to high volume centers to improve outcomes and decrease costs [4], there is a dearth of RPLND testis cancer volume outcomes studies. This is particularly important because this is a complex, potentially morbid operation that affects otherwise healthy young men. Our objectives are to characterize population-based RPLND utilization, costs, and inpatient outcomes and to assess the effect of hospital RPLND surgical volume on outcomes.

## 2. Materials and Methods

### 2.1. Study Cohort

We analyzed the US Healthcare Cost and Utilization Project (HCUP) Nationwide Inpatient Sample (NIS), from the Agency for Healthcare Research and Quality, between 2001 and 2008. NIS is a 20% stratified probability sample that encompasses hospitals in 42 states with approximately 8 million acute hospital stays from over 1000 hospitals per year. It is the largest all-payer inpatient care observational cohort in the US and represents approximately 90% of all hospital discharges.

Using International Classification of Diseases, 9th Edition (ICD-9) procedure codes, we identified RPLND (ICD-9 40.29, 40.3, 40.52, 40.59, 59.00) associated with a diagnosis of testis cancer (ICD-9 186x) in men aged ≥18 years during this period [5]. RPLND associated with laparoscopic (ICD-9 54.21, 54.51) and robotic (ICD-9 17.4x) approaches were identified, however, these 29 cases were excluded from the final cohort due to inability to power-adjusted analyses. We excluded subjects with hospital admissions for metastatic testis cancer: multiple sites of malignancy (ICD-9 196.x, 197.x, 198.x, 199.x, 202.x), chemotherapy administration (ICD-9 99.25, 99.28, V58.1x, E933.1), or autologous stem cell transplant (ICD-9 41.04). Moreover, because NIS does not characterize tumor stage, we excluded more complex RPLND performed concurrently with radical orchiectomy (ICD-9 62.3; *n* = 67), nephrectomy (ICD-9 55.5, 55.51; *n* = 27), or both orchiectomy and nephrectomy (*n* suppressed per NIS for 0 < *n* < 11) from our primary analysis to limit heterogeneity of comparison. However, as a subanalysis, we separately characterized differences in outcomes and costs by RPLND complexity.

### 2.2. Independent Variables

Due to the low incidence of testis cancer and RPLND, we used unweighted NIS sampling frequencies per HCUP recommendation. We examined patient (age, race, comorbidities [6], ZIP code-based median income, and primary payer) and hospital (US census region, urban versus rural location, teaching status, and bed size) level characteristics that may influence outcomes.

Of the 3726 hospitals sampled by the NIS from 2001–2008, 1469 (39%) treated adults with testis cancer, and 420 (11%) performed one or more RPLND. We categorized high volume hospitals *a priori *as those exceeding the 90th percentile in terms of annual procedural volume. With 33 of 62 hospitals performing only one RPLND that year, this resulted in a high volume designation of three or more RPLND versus a low volume designation of one to two RPLND. Additionally, we characterized volume as a continuous variable.

### 2.3. Outcomes

ICD-9 diagnosis and procedure codes were used to identify outcomes of interest: blood transfusions and complications (cardiac, respiratory, genitourinary, vascular, wound, miscellaneous medical, and miscellaneous surgical) [7]. (See the Supplementary Material S1 available online at: doi:10.1155/2012/189823.) NIS-specific outcomes included death, length of hospital stay (LOS), discharge disposition (routine (home) versus other (home healthcare, rehabilitation, skilled nursing facility, etc.)), and costs. Costs were derived from inpatient charges using the HCUP cost-to-charge ratio [8], and we quantified the effect of complications on hospital costs and LOS.

### 2.4. Statistical Analysis

Propensity score weighting [9, 10] was used to control for potential confounders among treatment groups, where the confounders included patient race, primary payer, income, and comorbidity, and hospital type, bed size, and geographic region. Each patient was weighted by the inverse propensity (or probability) of being in the treatment group, with the goal of balancing characteristics between groups. The propensity of being in each treatment group was calculated using multivariate logistic regression models based on the above potential confounders. All analyses were two sided and performed with SAS version 9.2 (SAS Institute Inc, Cary, North Carolina, U.S.A.) and were considered significant at *P* ≤ .05.

## 3. Results

### 3.1. Characteristics of Study Sample

We identified 993 men who underwent RPLND with a median age of 29 years (Table 1), the majority of whom was white (61.8%), with no or few comorbidities (85.8%) and were privately insured (71.4%). RPLND were mostly performed at urban teaching hospitals (79.6%) and large bed size facilities (78.7%).

512 (51.6%) of cases were performed at low volume hospitals where there were two or fewer RPLND per year. Men undergoing RPLND at high versus low volume hospitals were younger, more likely to be white and earning incomes exceeding the 50th percentile (Table 1, all *P* ≤ .05). High volume RPLND hospitals were more likely to be urban teaching hospitals with greater bed capacity (both *P* < .001). No differences were observed by hospital volume in terms of geographic region or patient comorbidity.

### 3.2. Outcomes

Unadjusted and propensity adjusted outcomes were similar. To demonstrate raw counts for clinical interpretability, we present unadjusted results by categorical volume with propensity adjusted *P* values (Table 2). While men undergoing RPLND at high versus low volume hospitals experienced fewer respiratory complications (4.2% versus 7.2%, *P* = .038), the frequency of cardiac, genitourinary, wound, vascular, miscellaneous medical, and surgical complications, and transfusion rates were similar using the dichotomous volume definition. RPLND inpatient mortality was less than  .02%, median LOS was five days, most men were routinely discharged home (97.8%), and the median cost of RPLND was $10,490. While mortality, LOS, and discharge status did not vary by dichotomous hospital volume, median RPLND costs were greater at high versus low volume hospitals ($11,365 versus $9,930, *P* < .001).

When outcomes were assessed by hospital volume as a continuous variable, more notable differences were observed (Table 3). For instance, higher hospital volume was associated with fewer respiratory, miscellaneous medical complications and overall complications (all *P* ≤ .047). While higher hospital volume was associated with a greater likelihood for routine home discharge (*P* = .017), it was also associated with more blood transfusions (*P* = .004) when volume was assessed as a continuous rather than dichotomous variable. There was no hospital volume relationship with LOS, however, higher costs were associated with increasing RPLND volume (*P* < .001).

While transfusions and most complications extended LOS, this was most pronounced for vascular and miscellaneous medical complications, which prolonged hospitalization by two and 2.5 days, respectively (both *P* ≤ .021, Table 4). Additionally, most complications were associated with a lower likelihood of routine home discharge. In particular, wound complications decreased routine discharge by 17.3% (*P* < .001). Overall, complications increased median hospitalization costs by $2,784, and all complications aside from wound complications were associated with higher costs. Most notably, cardiac and vascular complications increased hospital costs by $6,322 and $6,405, respectively, and transfusions increased costs by $5,933 (all *P* < .001).

### 3.3. RPLND with Concurrent Operation

RPLND with concurrent operations was associated with more complications and transfusions, particularly RPLND with nephrectomy (both *P* < .001, Table 5). Men undergoing RPLND with concurrent orchiectomy and nephrectomy experienced greater mortality and fewer routine discharges (both *P* < .001). Similarly, LOS increased with additional procedures (*P* < .001). Costs were also higher with additional procedures, particularly for concurrent nephrectomy, with increments ranging from $14,074 to $19,697 over RPLND alone (*P* < .001).

## 4. Discussion

The relative low incidence of testis cancer (5.5 per 100,000 men) compared with more common genitourinary cancers such as prostate cancer (156 per 100,000 men) [11] contributes to the challenge of exploring RPLND population-based outcomes. While published RPLND outcomes come largely from experienced high volume hospitals [12–17], we found that more than half of all RPLND are performed at hospitals with volumes of less than three RPLND annually. Thus, with a more stringent definition for “high volume” of >40 cases per year, the vast majority of RPLND is performed at low volume centers. Therefore published RPLND series may not be representative of community outcomes. While higher provider volume is associated with improved outcomes for genitourinary oncologic conditions including radical prostatectomy, cystectomy, and nephrectomy [4], this effect has yet to be studied or demonstrated for RPLND. To our knowledge, this is the first population-based study to characterize RPLND patterns of care, outcomes, and costs, and to demonstrate volume outcomes effects. 

Our study has several important findings. First, sociodemographic differences were observed between high versus low volume hospitals. While a previous population-based study demonstrated that RPLND utilization did not vary by socioeconomic or racial backgrounds [3], our study demonstrates that nonwhites with lower incomes were less likely to undergo RPLND at high volume hospitals. For other urologic cancers, higher provider volume is associated with better outcomes and lower mortality [4]. Similarly, several studies demonstrate improved survival for men with metastatic testis cancer who were treated with chemotherapy at higher volume centers [18–20]. This raises concern whether minorities and lower income men have unequal access to high volume centers and the comprehensive cancer care they offer, which has been shown to be disparate for several other complex surgical procedures [21].

Second, while higher hospital volumes were associated with fewer respiratory complications when assessing volume dichotomously, RPLND volume assessed as a continuous variable was associated with fewer overall and miscellaneous medical complications, and more routine home discharges. Unlike more common procedures where higher volume, defined in most series as ≥66th percentile, has been associated with improved outcomes [22–24], even a 90th percentile volume appears to be too low a threshold for RPLND in that significant volume effects are demonstrated only at higher volume extremes for a procedure as uncommon as RPLND. However, this is noted in the context where higher volume centers are more likely to transfuse and incur higher costs. A likely explanation for this may be that high volume referral centers are more likely to treat complex cases, including postchemotherapy and salvage resections. RPLND with concurrent orchiectomy and/or nephrectomy are more likely to be associated with these complex case scenarios, and are referred to high volume hospitals, and thus pose greater risks for complications. Our study likely underestimates RPLND volume outcomes effects due to our inability to adjust for tumor stage and prior chemotherapy use to differentiate case complexity.

Third, increased costs were associated with higher volume hospitals and concurrent operations. This may also be related to the greater complexity of cases treated at high volume centers, which are usually academic and focus on research, teaching, and patient care that is irrespective of the clinical or financial risks [25]. Higher costs may also be attributable to superior information technology and documentation of high volume hospitals, which may lead to improved compliance with guidelines to improve reimbursements. It has also been shown that hospitals with significant market shares are able to negotiate more competitive prices with insurers that could lead to higher costs [26].

Fourth, RPLND involving additional procedures, particularly the use of nephrectomy (3%), were associated with significantly more transfusions, complications, nonroutine discharges, longer LOS, and higher hospital costs. It is noteworthy that while high volume hospital cases required more transfusions, which are associated with a cost increase of nearly $6,000 more per case, the overall differential over low volume hospitals was only $1,400, suggesting that higher volume may also offset costs associated with increased case complexity and adverse outcomes. Despite these increased costs, these estimates are lower than previously reported high volume hospital costs, where modified RPLND averaged $16,900, and postchemotherapy RPLND averaged $28,600 [27].

Finally, complications and transfusions were associated with increased LOS and costs, and fewer routine discharges. While transfusions and cardiac and vascular complications incurred the greatest cost increases, wound complications yielded only $2,875 more in hospitalization costs but had the greatest negative effect on routine discharge. It is likely that additional costs are passed onto skilled nursing facilities that are not captured beyond the inpatient scope of NIS. However, our assessment of RPLND costs and outcomes are relevant to the current U.S. healthcare debate. The recent Medicare initiative to utilize “spending per beneficiary” as a measure of hospital performance aims to incentivize hospitals that incur fewer complications and lower costs [28]. For RPLND and other complex operations, higher risk patients and those with greater case complexity are treated at referral centers, which incur higher costs despite better clinical outcomes. This policy raises concern that hospitals that treat higher risk patients may be penalized under this paradigm, though Medicare aims to adjust for disease severity.

Our study must be interpreted within the context of the study design. First, administrative data are designed for billing purposes and may lack detailed clinical information; we were unable to characterize tumor characteristics, disease severity, and thus could not distinguish between primary versus postchemotherapy and salvage RPLND, which may affect patient selection and outcomes. While we minimized heterogeneity by excluding those admitted for metastatic disease or undergoing concurrent operations from our primary RPLND analyses, differences in case complexity remains a potential confounder. However, administrative data are accurate in capturing surgical complications [29], and outcomes are standardized in contrast to comparison of case series, which may vary in defining complications and have observer bias in recording outcomes. Second, there is missing race data for 21.7% of subjects. This may reflect differences in actual patient demographics, whereby nonwhite minority designations may not be specified, or may reflect systematic differences in race identification between low and high volume hospitals. Third, NIS does not contain surgeon level characteristics and we were unable to adjust for surgeon experience. Fourth, NIS is limited to the inpatient setting, and we were unable to assess long-term oncologic outcomes such as recurrence and subsequent chemotherapy use, outpatient complications, and costs. Finally, this is an observational study, and there may be additional unobserved confounders that we were unable to adjust for.

## 5. Conclusion

This is the first population-based study of RPLND utilization, costs and outcomes that also characterizes hospital volume outcomes effects for RPLND. The majority of cases are performed at hospitals with two or fewer RPLNDs per year. Sociodemographic differences exist between high versus low RPLND volume hospitals, which suggests an inequity in access. Higher volume hospitals had higher transfusion rates and incurred higher costs, possibly related to higher degrees of surgical case complexity. Finally, high volume hospitals had fewer complications and greater likelihood of routine home discharge, despite likely greater case complexity.

## Supplementary Material

ICD-9 codes for blood transfusion and complications.

## Figures and Tables

**Table 1:** Characteristics of the RPLND study population.

	Total	Hospital volume		*P* value
		Low	High	
	*n* = 993	*n* = 512	*n* = 481	
Age				
Mean (SD)	30.9 (9.4)	31.7 (9.7)	30.4 (9.4)	.006
Median (IQR)	29.0 (23.0–36.0)	31.0 (24.0–38.0)	27.0 (23.0–35.0)	.003
Range	18–78	18.0–78.0	18.0–66.0	
Race (*n*/%)				
White	614 (61.8)	283 (55.3)	331 (68.8)	<.001
Non-White	164 (16.5)	83 (16.2)	81 (16.8)	
Missing	212 (21.7)	145 (28.5)	69 (14.4)	
Primary Payer (*n*/%)				
Private	709 (71.4)	370 (72.3)	339 (70.5)	.017
Medicare	38 (3.8)	11 (2.2)	27 (5.6)	
Medicaid/Other	246 (24.8)	131 (25.6)	115 (23.9)	
Income Quartile (*n*/%)				
$1–$38,999	154 (21.4)	72 (20.8)	82 (21.7)	.050
$39,000–$47,999	173 (23.9)	98 (28.2)	75 (19.8)	
$48,000–$62,999	195 (26.9)	83 (23.9)	112 (29.6)	
$63,000+	203 (28.0)	94 (27.1)	109 (28.8)	
Hospital Type (*n*/%)				
Rural	20* (2)	20* (3)	0	<.001
Urban Non-Teaching	180* (18)	180* (35)	DS (2)	
Urban Teaching	790* (80)	320* (62)	470* (98)	
Hospital Bed Size (*n*/%)				
Small	88 (8.9)	41 (8.0)	47 (9.8)	<.001
Medium	124 (12.5)	93 (18.2)	31 (6.4)	
Large	781 (78.7)	378 (73.8)	403 (83.8)	
Hospital Region (*n*/%)				
Northeast	186 (18.7)	102 (19.9)	84 (17.5)	.337
Midwest	246 (24.8)	128 (25.0)	118 (24.5)	
South	325 (32.7)	172 (33.6)	153 (31.8)	
West	236 (23.8)	110 (21.5)	126 (26.2)	
Comorbidity (*n*/%)				
None	452 (45.5)	241 (47.1)	211 (43.9)	.076
One	400 (40.3)	190 (37.1)	210 (43.7)	
Multiple	141 (14.2)	81 (15.8)	60 (12.5)	

Abbreviations: SD: standard deviation, IQR: interquartile range, DS: data suppressed per NIS for 0 < *n* < 11.

*n rounded to nearest 10 to prevent calculation of suppressed data, % based on rounded.

*n*, numbers may not add up to total and % may not add up to 100 due to rounding.

**Table 2:** RPLND outcomes and costs by dichotomous hospital volume.

	Total	Hospital volume		*P* value
		Low	Low	
	*n* = 993	*n* = 512	*n* = 481	
Complications (*n*/%)				
Cardiac	DS (0)*	DS (0)*	DS (0)*	.678
Respiratory	57 (5.7)	37 (7.2)	20 (4.2)	.040
Genitourinary	28 (2.8)	16 (3.1)	12 (2.5)	.091
Wound	27 (2.7)	16 (3.1)	11 (2.3)	.339
Vascular	DS (0)*	DS (0)*	DS (0)*	.631
Miscellaneous Medical	133 (13.4)	70 (13.7)	63 (13.1)	.286
Miscellaneous Surgical	51 (5.1)	27 (5.3)	24 (5.0)	.506
Death	DS (0)*	DS (0)*	0	.116
Any complication	246 (24.8)	138 (27.0)	108 (22.5)	.246
Blood Transfusion (*n*/%)	34 (3.4)	11 (2.2)	23 (4.8)	.064
Routine Discharge (*n*/%)	971 (97.8)	498 (97.3)	473 (98.3)	.315
LOS (Median days/IQR)	5.0 (4.0–6.0)	5.0 (4.0–6.0)	5.0 (4.0–7.0)	.391
Costs (Median/IQR)	$10,490 ($7,792–$14,969)	$9,930 ($6,933–$12,927)	$11,365 ($8,394–$18,402)	<.001

Abbreviations: DS: data suppressed per NIS for 0 < *n* < 11, LOS: length of stay, IQR: interquartile range.

*% rounded to nearest 5 to prevent calculation of suppressed data.

**Table 3:** RPLND outcomes by continuous hospital volume.

	*n*	Hospital volume		*P* value
		Median (IQR)	Mean (SD)	
Complications				
Cardiac				
Y	DS	3.0 (1.0–5.0)	3.3 (2.4)	.788
N	980*	2.0 (1.0–6.5)	14.3 (27.9)	
Respiratory				
Y	57	2.0 (1.0–3.0)	6.4 (16.3)	.047
N	936	2.0 (1.0–6.5)	14.7 (28.2)	
Genitourinary				
Y	28	2.0 (1.0–5.0)	12.3 (27.9)	.615
N	965	2.0 (1.0–6.5)	14.3 (27.8)	
Wound				
Y	27	2.0 (1.0–5.0)	5.5 (12.0)	.483
N	966	2.0 (1.0–6.5)	14.5 (28.0)	
Vascular				
Y	DS	1.0 (1.0–64.0)	23.7 (31.3)	.446
N	990*	1.0 (1.0–6.5)	14.2 (27.7)	
Miscellaneous Medical				
Y	133	2.0 (1.0–6.0)	6.1 (14.0)	.027
N	860	2.0 (1.0–6.5)	15.5 (29.1)	
Miscellaneous Surgical				
Y	51	2.0 (1.5–3.0)	4.5 (12.5)	.468
N	942	2.0 (1.0–6.5)	14.7 (28.3)	
Any complication				
Y	246	2.5 (1.0–5.0)	7.0 (16.9)	.004
N	747	2.0 (1.0–7.0)	16.6 (30.1)	
Blood Transfusion				
Y	34	4.5 (1.5–13.0)	22.2 (33.8)	.004
N	959	2.0 (1.0–6.5)	13.9 (27.5)	
Routine Discharge				
Y	22	2.0 (1.0–6.5)	14.5 (28.0)	.017
N	971	1.5 (1.0–3.0)	2.0 (1.4)	

Abbreviation: IQR: Interquartile range, SD: Standard deviation, DS: Data suppressed per NIS for 0 < *n* < 11.

*n rounded to nearest 10 to prevent calculation of suppressed data.

**Table 4:** Length of stay, discharge status, and costs by clinical outcomes.

	*n*	LOS (Median days/IQR)	*P* value	Routine discharge (*n*/%)	*P*-value	Cost (median/IQR)	*P* value
Complications							
Cardiac							
Y	DS	6.0 (4.0–8.0)	.084	DS (100)	1.000	$16,746 ($13,283–$28,343)	.011
N	980*	5.0 (4.0–6.0)		962 (97.8)		$10,424 ($7,747–$14,778)	
Respiratory							
Y	57	6.0 (5.0–8.0)	<.001	53 (93.0)	.033	$12,897 ($10,072–$18,276)	.001
N	936	5.0 (4.0–6.0)		918 (98.1)		$10,360 ($7,695–$14,602)	
Genitourinary							
Y	28	5.5 (4.0–9.0)	.027	27 (96.4)	.471	$12,883 ($10,358–$19,192)	.021
N	965	5.0 (4.0–6.0)		944 (97.8)		$10,394 ($7,738–$14,798)	
Wound							
Y	27	6.0 (4.0–15.0)	.002	22 (81.5)	<.001	$13,268 ($6,715–$34,577)	.412
N	966	5.0 (4.0–6.0)		949 (98.2)		$10,483 ($7,799–$14,788)	
Vascular							
Y	DS	7.0 (7.0-8.0)	.021	DS (100)	1.000	$16,823 ($10,948–$22,227)	.047
N	990*	5.0 (4.0–6.0)		965 (97.8)		$10,418 ($77,88–$14,807)	
Miscellaneous Medical							
Y	133	7.0 (5.0–9.0)	<.001	126 (94.7)	.020	$12,634 ($9,792–$18,276)	<.001
N	860	4.5 (4.0–6.0)		845 (98.3)		$10,168 ($7,608–$14,407)	
Miscellaneous Surgical							
Y	51	6.0 (5.0–8.0)	<.001	47 (92.2)	.023	$13,285 ($8,690–$24,662)	.002
N	942	5.0 (4.0–6.0)		924 (98.1)		$10,392 ($7,725–$14,611)	
Any complication							
Y	246	6.0 (5.0–8.0)	<.001	234 (95.1)	.001	$12,689 ($9,537–$18,814)	<.001
N	747	4.0 (3.0–6.0)		737 (98.7)		$9,905 ($7,558–$13,831)	
Blood Transfusion							
Y	34	6.0 (5.0–8.0)	<.001	32 (94.1)	.172	$16,293 ($16,293–$28,717)	<.001
N	959	5.0 (4.0–6.0)		939 (97.9)		$10,360 ($7,696–$14,424)	

Abbreviation: LOS: length of stay, IQR: interquartile range, DS: data suppressed per NIS for 0 < *n* < 11.

*n rounded to nearest 10 to prevent calculation of suppressed data.

**Table 5:** Variation in RPLND complexity and outcomes.

	RPLND	RPLND + orchiectomy	RPLND + nephrectomy	RPLND + Orchiectomy + nephrectomy	*P* value
	*n* = 993	*n* = 67	*n* = 27	*n* = DS	
Death (*n*/%)	DS (0)*	0	0	DS (15)*	<.001
Any Complication (*n*/%)	246 (24.8)	24 (35.8)	17 (63.0)	DS (30)*	<.001
Blood Transfusion (*n*/%)	34 (3.4)	DS (5)*	DS (20)*	DS (55)*	<.001
Routine Discharge (*n*/%)	971 (97.8)	65 (97.0)	25 (92.6)	DS (70)*	<.001
LOS (Median days/IQR)	5 (4–6)	6 (4–8)	8 (6–10)	9 (6–15)	<.001
Costs (Median/IQR)	$10,490 ($7,792–$14,969)	$15,478 ($9,799–$25,340)	$30,187 ($14,100–$37,034)	$24,564 ($21,761–$43,698)	<.001

Abbreviations: DS; data suppressed per NIS for 0 < *n* < 11, LOS; length of stay, IQR; interquartile range.

*% rounded to nearest 5 to prevent calculation of suppressed data.

## Appendix

See the Supplementary Material S1 available online at: doi:10.1155/2012/189823.
