# Supplementary material for: Hospital Surgical Volume, Utilization, Costs and Outcomes of Retroperitoneal Lymph Node Dissection for Testis Cancer
Source: Adv Urol. 2012 Apr 9;2012:189823. doi: 10.1155/2012/189823 (PMC3328891; doi:10.1155/2012/189823)
Supplement: Supplementary file 1 — ICD-9 codes for blood transfusion and complications. [file 189823.f1.pdf]

Supplementary Table S1.

ICD-9 codes for blood transfusion and complications.

Category	Diagnosis codes	Procedure codes
Blood Transfusion	V58.2	99.00, 99.02, 99.03, 99.04
Cardiac	410.xx, 402.01, 402.11, 402.91, 428.xx, 427.5, 997.1	
Respiratory	518.0, 514, 518.4, 466.xx, 480.xx, 481, 482.xx, 483.xx, 485, 486, 518.5, 518.81, 518.82, 799.1, 997.3	
Genitourinary	590.1x, 590.2, 590.8x, 590.9, 591, 593.3, 593.4, 593.5, 593.81, 593.82, 595.89, 596.1, 596.2, 596.6, 997.5	55.02, 55.03, 55.12, 55.93, 55.94, 59.93, 97.61, 97.62, 56.1, 56.41, 56.74, 56.75, 56.81, 56.84, 56.86, 56.89, 56.91
Wound	567.xx, 998.3, 998.5x, 998.6	54.61, 54.1x, 54.91, 54.0, 59.19
Vascular	415.1, 451.1x, 451.2, 451.81, 451.9, 453.8, 453.9, 997.2, 999.2, 444.22, 444.81, 433.xx, 434.xx, 436, 437.xx	
Miscellaneous medical	584.xx, 586, 785.5x, 995.0, 995.4, 998.0, 999.4, 999.5, 999.6, 999.7, 999.8, 457.8, 560.1, 560.8x, 560.9, 997.4, 353.0, 354.2, 723.4, 955.1, 955.3, 955.7, 955.8, 955.9, 531.xx, 532.xx, 533.xx, 782.4, 573.8	
Miscellaneous surgical	599.1, 596.1, 596.6, 565.1, 569.3, 569.83, 569.4x, 998.1x, 998.83, 998.9, 998.2, 998.4, 998.7, 604.0, 956.0, 956.1, 956.4, 956.5, 956.8, 956.9, 902.50, 902.51, 902.52, 902.53, 902.54, 902.59	46.03, 46.04, 46.10, 46.11, 46.14, 48.4x, 48.5, 48.6x, 48.7x, 48.9x
